# *L. reuteri* ZJ617 inhibits inflammatory and autophagy signaling pathways in gut-liver axis in piglet induced by lipopolysaccharide

**DOI:** 10.1186/s40104-021-00624-9

**Published:** 2021-10-13

**Authors:** Tao Zhu, Jiangdi Mao, Yifan Zhong, Congxiang Huang, Zhaoxi Deng, Yanjun Cui, Jianxin Liu, Haifeng Wang

**Affiliations:** 1 The Key Laboratory of Molecular Animal Nutrition, Ministry of Education, College of Animal Science, Zhejiang University, Hangzhou, 310058 China; 2 Xixi Hospital of Hangzhou, Hangzhou, 310023 China

**Keywords:** Gut-liver axis, Hepatic injury, Intestinal barrier, *Lactobacillus*, Piglet, Signaling pathways

## Abstract

**Background:**

This study investigated the protective effects of *L. reuteri* ZJ617 on intestinal and liver injury and the underlying mechanisms in modulating inflammatory, autophagy, and apoptosis signaling pathways in a piglet challenged with lipopolysaccharide (LPS).

**Methods:**

Duroc × Landrace × Large White piglets were assigned to 3 groups (*n* = 6/group): control (CON) and LPS groups received oral phosphate-buffered saline for 2 weeks before intraperitoneal injection (i.p.) of physiological saline or LPS (25 μg/kg body weight), respectively, while the ZJ617 + LPS group was orally inoculated with ZJ617 for 2 weeks before i.p. of LPS. Piglets were sacrificed 4 h after LPS injection to determine intestinal integrity, serum biochemical parameters, inflammatory signaling involved in molecular and liver injury pathways.

**Results:**

Compared with controls, LPS stimulation significantly increased intestinal phosphorylated-p38 MAPK, phosphorylated-ERK and JNK protein levels and decreased IκBα protein expression, while serum LPS, TNF-α, and IL-6 concentrations (*P* < 0.05) increased. ZJ617 pretreatment significantly countered the effects induced by LPS alone, with the exception of p-JNK protein levels. Compared with controls, LPS stimulation significantly increased LC3, Atg5, and Beclin-1 protein expression (*P* < 0.05) but decreased ZO-1, claudin-3, and occludin protein expression (*P* < 0.05) and increased serum DAO and D-xylose levels, effects that were all countered by ZJ617 pretreatment. LPS induced significantly higher hepatic LC3, Atg5, Beclin-1, SOD-2, and Bax protein expression (*P* < 0.05) and lower hepatic total bile acid (TBA) levels (*P* < 0.05) compared with controls. ZJ617 pretreatment significantly decreased hepatic Beclin-1, SOD2, and Bax protein expression (*P* < 0.05) and showed a tendency to decrease hepatic TBA (*P* = 0.0743) induced by LPS treatment. Pretreatment of ZJ617 before LPS injection induced the production of 5 significant metabolites in the intestinal contents: capric acid, isoleucine 1TMS, glycerol-1-phosphate byproduct, linoleic acid, alanine-alanine (*P* < 0.05).

**Conclusions:**

These results demonstrated that ZJ617 pretreatment alleviated LPS-induced intestinal tight junction protein destruction, and intestinal and hepatic inflammatory and autophagy signal activation in the piglets.

**Supplementary Information:**

The online version contains supplementary material available at 10.1186/s40104-021-00624-9.

## Introduction

*L. reuteri* species are an obligate heterofermentative strain that exerts beneficial effects on the health of the host [1]. It was first isolated in 1962 and can be found endogenously in the intestines of all vertebrates and mammals [2], although often in relatively low numbers. *L. reuteri* has great potential to exert its probiotic effects given its tolerance to the gastrointestinal environment and the secretion of special bacteriostatic substances such as reuterin which is produced by glycerol metabolism [3, 4]. Based on the strong adhesion and broad-spectrum antibacterial activity of reuterin, there have been numerous studies investigating the relationship between the effects of *L. reuteri* and intestinal health [5–7]. The intestine is an important organ that exerts numerous functions, including the digestion and absorption of various nutrients [8], resistance to noxious exogenous factors [9], and activation of transduction of signaling pathways related to innate immunity and adaptive immunity [10]. The lumen of the gut is always exposed to a plethora of microorganism, food antigens, and toxins, thus it is susceptible to triggering immune responses and inducing intestinal dysfunction and dysbiosis, which ultimately lead to various intestinal diseases, such as irritable bowel syndrome (IBS) [11] and diarrhea [12]. The intestinal epithelial barrier formed by the intestinal mucosa is vulnerable to damage when the gut is subjected to external stress like lipopolysaccharides (LPS) [13] or pathogenic bacteria [14].

Many studies have shown that *Lactobacillus spp*. can alleviate damage to the intestinal barrier. *L. acidophilus* has been shown to normalize the expression of tight junction (TJ) proteins (occludin and claudin-1) and prevented interleukin (IL)-1β-induced NF-κB activation in Caco-2 cells [15]. The administration of *L. rhamnosus* GG (LGG) could improve intestinal barrier function in piglets challenged with LPS [16]. *In vitro* experiments have demonstrated that *Lactobacillus spp.* fortified intestinal barrier function and preserved TJ integrity, but no beneficial effects were observed *in vivo* when given alone [17]. *L. reuteri* LR1 supplementation improved intestinal morphology and intestinal barrier function in weaned piglets [5]. Oral inclusion of *L. reuteri* FN041 for 4 weeks improved intestinal epithelial barrier function in mice after a 7-week high-fat diet [6].

Intestinal barrier injury is often accompanied by the activation of the immune system and an inflammatory response [18]. Previous studies have shown that *Lactobacillus spp.* has immunomodulatory and anti-inflammatory effects and recent studies have focused on the underlying mechanisms. Several strains of *L. reuteri* alleviated inflammation by reducing the production of pro-inflammatory cytokines. *L. reuteri* GMNL-263 lowered serum MCP-1, tumor necrosis factor (TNF)-α, and IL-6 levels in mice fed with a high-fat diet [19]. Similar effects were observed in mice treated with *L. reuteri* 6475 [7] and ZJ617 culture supernatants [20], indicating that culture supernatants could exert similar anti-inflammatory activity in the gut as probiotics.

A growing body of evidence has emerged supporting the view that LPS generates reactive oxygen species (ROS), which activate NF-κB and induce inflammation [21]. Malondialdehyde (MDA) is one of the most important products of membrane lipid peroxidation and is used to indirectly measure the degree of damage to the membrane system [22]. Glutathione peroxidase (GSH-Px) is frequently used as an index of lipid peroxidation [23]. Superoxide dismutase (SOD) is a critical antioxidant metal enzyme that balances oxidation and antioxidation [24]. In order to fully understand the upstream and downstream conditions of the inflammatory pathway, data regarding alterations of the oxidative stress pathway are required.

As an intracellular lysosome-dependent degradation system, autophagy plays an essential role in maintaining homeostasis when cells face starvation and other forms of stress insults. In order to maintain homeostasis, autophagy destroys damaged proteins and organelles after an LPS-challenge [25]. A study has shown that LGG suppressed autophagy in *Salmonella*-challenged pigs [26]. *L. rhamnosus* effectively recovered autophagic flux and attenuated the inflammation in Caco-2 cells impaired by *F. nucleatum* [27]. *L. reuteri* 100–23, *L. gasseri* IPL A6.33, *L. rhamnosus* IPL A2.21, *L. gasseri* CMUL057 and *L. acidophilus* CMUL067 are able to induce autophagy activation to release the anti-inflammatory cytokine interleukin-10 and inhibit the secretion of the pro-inflammatory cytokine interleukin-1ß [28]. These results suggested *Lactobacillus* was able to interfere with autophagy. Therefore, it is important to investigate the influence of oral inclusion of ZJ617 on intestinal autophagy. Moreover, growing literature has shown that there is a crosstalk between autophagy and inflammation in the intestine [29], while autophagy also mediates pivotal functions in innate and adaptive immunity [30].

As one of the most important detoxification organs of human body, the liver plays a pivotal role following infections or when exposed to toxins. Through the portal vein system, there is a close anatomical and functional association between the liver and the intestine. Most liver diseases and its complications are mediated by the gut-liver axis [31, 32]. Many animal studies have revealed that gut microbiota dysbiosis induces hepatic injury due to increased gut permeability, which contributes to increased hepatic exposure to harmful substances [33, 34]. Since the intestinal flora can be modulated to affect the liver, a growing number of studies are emerging investigating whether the supplementation of probiotics could improve liver diseases. Evidence has shown that *Lactobacillus spp.* play an important role in alleviating liver damage [35]. The levels of pro-inflammatory cytokines (IL-1β, IL-6, and TNF-α) in the liver were significantly reduced in T2D mice after oral administration of *L. acidophilus* KLDS1.1003 and KLDS1.0901 for 6 weeks [36]. The protein expression of hepatic IL-1β, IL-6, and TNF-α was reduced after supplementation with *L. aracasei* GMNL-32, *L. reuteri* GMNL-89, and *L. reuteri* GMNL-263 by suppressing MAPK and NF-κB signaling pathways [37]. The same effect was observed in a model of cholestatic liver disease in mice, the hepatic gene expression of IL-6 was reduced by probiotic LGG [38].

A tight bidirectional crosstalk existed between the gut and liver, the so-called “gut-liver axis”, via the biliary tract, portal vein and systemic circulation [39]. For instance, bile acids are molecules synthesized from cholesterol in the liver and then are released to the gastrointestinal tract where they are involved in digestion and are metabolized by intestinal flora. Circulation of bile acids depends on an active feedback loop between the liver and the intestine. Studies have shown that LPS induced higher serum levels of total bile acid (TBA) in mice for the synthesis of bile acids when the liver was damaged [40]. In addition, evidence has demonstrated that bile acids were also involved in the inflammatory response of macrophages [41]. When the intestinal barrier function is disrupted, an increase in intestinal permeability leads to the translocation of bacteria-derived components including LPS to the liver via the portal vein, which stimulate hepatic innate immune receptors to induce the acute and chronic inflammatory response [42].

Previously, we revealed that ZJ617 could modulate intestinal immune responses and metabolism in LPS-stimulated mice [43]. We found that ZJ617 could protect intestinal barrier dysfunction in mice via antioxidant activities and enhancing TJ expression [44]. Based on these previous studies, we hypothesized that ZJ617 could relieve intestinal barrier injury and reduce levels of inflammatory cytokines, and further alleviate the damage to the liver in LPS-challenged piglets. This study was aimed to investigate whether ZJ617 has a protective effect on intestinal and liver injury in LPS-challenged piglets and to explore the underlying mechanisms involved by evaluating inflammatory and autophagy signaling pathways.

## Methods

### Culture of ZJ617 and preparation of freeze-dried power

ZJ617 was previously isolated from piglet intestine and kept in our laboratory. The strain was anaerobically cultured for 18 h in sterile De Man Rogosa and Sharpe (MRS) medium at 37 °C. During the logarithmic growth period, the bacterial culture was harvested and then centrifuged at 4000 × *g* for 5 min at 4 °C. ZJ617 was suspended in skimmed milk and freeze-dried to form powder in vacuum for 14 h. The strain powder was stored in sealed packets at 4 °C, measured by plate count, and found to contain 2.5 × 10^10^ CFU/g.

### Animals studies

This research was specifically approved by the Animal Care and Use Committee of Zhejiang University (ethics code permit no. ZJU 20170529). The basal diet was formulated without antibiotics based on recommendations of the NRC. Eighteen healthy weaned piglets (Duroc × Landrace × Large White), including males and females, of similar body weight (age: 28 ± 0 d; mean body weight: 8.6 ± 1.1 kg), were divided into 3 groups: control (CON), LPS, and ZJ617 + LPS. Piglets in the ZJ617 + LPS group were orally inoculated with ZJ617 strain dissolved in PBS (1 × 10^10^ CFU/d) for 2 weeks. Piglets in the CON and LPS groups were orally inoculated with PBS for 2 weeks. At 14 d after the initiation of the oral inoculation, the LPS and ZJ617 + LPS group piglets were intraperitoneally injected with 25 μg/kg LPS of *E. coli* serotype 055:B5(Sigma-Aldrich), while the CON group piglets were i.p. injected with physiological (0.9%) saline.

Four hours after LPS challenge, rectal temperatures were detected and piglets were killed by intravenous injection of sodium pentobarbital (200 mg/kg body weight). Blood, distal ileum and ilea content samples were collected for assays. Serum samples were obtained by centrifuging the blood at 3000 × *g* for 15 min at 4 °C. Segments (1 cm × 1 cm) of the distal ileum were flushed gently with 5 mL 0.9% saline twice for use in histopathological and Western blotting analyses. The luminal contents of the ileum were also collected and stored at − 80 °C.

### Biochemical assays for the serum and liver tissues

Concentrations of TNF-α, IL-6, and IL-10 in the serum were measured using ELISA with commercially available kits (nos. H052, H007, and H009, respectively; Nanjing Jiancheng Bioengineering Institution). Activities of alanine aminotransferase (ALT; no.C009), aspartate aminotransferase (AST; no.C010), superoxide dismutase (SOD; no.A001–1-1), glutathione peroxidase (GSH-Px; no.A005–1-1), lactate dehydrogenase (LDH; no.A020–2-2), malondialdehyde (MDA; no.A003–1-1) myeloperoxidase (MPO; no.A044–1-1) and diamine oxidase (DAO; no.A088) activity and D-xylose (no.A035) concentrations in the serum were determined by kinetics-based assays with commercially available kits (Nanjing Jiancheng Bioengineering Institution) followed by analyses on an automatic biochemistry analyzer (SELECTA XL; Vital Scientific) according to a protocol provided by the manufacturer. LPS levels were measured with endotoxin detection limulus kit (Xiamen Bioendo Technology,) according to the manufacturer’s instructions.

### Western blotting analysis

Ileal tissues and liver tissues were lysed using a lysis buffer (Sigma, USA). Total protein concentration was determined using the BCA method. Western blotting analysis was performed as previously described [44]. The primary antibodies included rabbit anti-IκBα, anti-GAPDH, anti-p38, anti-phospho-p38 (p-p38), anti-JNK, anti-phospho-JNK (p-JNK), anti-extracellular signal-regulated kinase (anti-ERK), anti-p-ERK, anti-Atg5, anti-Beclin 1, anti-SOD2, anti-Bax, anti-claudin-3, anti-occludin, anti-zonula occludens 1 (ZO-1), anti-LC3 (Cell Signaling Technology, USA; 1:1000). The second antibody was HRP, goat anti-rabbit IgG (Abbkine, Beijing; 1:5000).

Specific proteins were detected using an enhanced chemiluminescence kit (Perkin Elmer Life Sciences, USA). Protein bands were visualized with a chemiluminescence substrate and a gel-imaging system (Tanon Science and Technology, shanghai) and analyzed with Image Analysis software (NIH, USA). In all instances, the density values of bands were corrected after subtracting background values. GAPDH was used as the internal reference protein.

### Immunohistochemical analysis

Sections of distal ileal samples were prepared by the method described previously [45]. Immersing tissue samples of appropriate size in 4% formalin for 1 d at room temperature. The fixed tissues were trimmed and embedded in paraffin, and cut into slices of 4 μm thickness. After paraffin removal and rehydration, the sections were blocked with 10% normal goat serum for 1 h. Firstly, primary rabbit antibody (anti-p38, anti-p-p38, anti-ERK, anti-p-ERK, anti-ZO-1, anti-claudin-3, or anti-occludin; 1:1000; Cell Signaling Technology) were used to incubate the sections for one night at 4 °C. Then, horseradish peroxidase (HRP)-conjugated secondary antibody was used for 1 h. By using the diaminobenzidine-HRP detection system, sections were counterstained with hematoxylin, dehydrated, and covered with cover slips. A microscope (ECLIPSE Ti; Nikon Corporation) was used to assess the immunostaining and analyzed using Image-Pro Plus 6.0 software (Media Cybernetics). Positive cells were stained brown.

### Metabolomic analysis

According to the method described previously [43], the supernatant (0.30 mL) were extracted from ileal contents of each sample and centrifuged at 13,300 × *g* for 15 min at 4 °C. A 7890 Gas Chromatograph System (Agilent Technologies) and a Pegasus™ HT TOF MS(LECO) were used to analyze samples. Extraction of raw peaks, filtering and calibration of the baselines, peak alignment, deconvolution analyses, peak identification, and integration of the peak area were performed by Chroma TOF 4.3X (LECO) and the X Rtx5database (LECO). The retention time index (RI) was used for peak identification, with an RI tolerance of 5000. Metabolic features detected in < 50% of quality-control samples were removed.

Data were subjected to multivariate analysis by partial least squares-discriminant analysis (PLS-DA) and orthogonal projections to latent structures-discriminant analysis (OPLS-DA) with the use of SIMCA-P v13.0 (Umetrics, Umea, Sweden) [46]. The PLS-DA model was validated by 200 permutation tests and used to obtain a higher level of data separation. OPLS-DA was undertaken to obtain maximal covariance among the data. The first principal component of the variable in importance projection (VIP) was obtained to refine the analysis. Variable metabolites were selected at VIP > 1.5 and then assessed by the student’s *t*-test. Variables between the two comparison groups were discarded if *P* > 0.05 [7]. The fold change (FC) of metabolites was obtained by comparing mean peak values between each of the two groups. Obtained differential metabolites were further identified and validated by searching in the Kyoto Encyclopedia of Genes and Genomes (KEGG). Each differential metabolite was then cross listed with the pathways in the KEGG, and top altered pathways were identified and finally used for the potential functional analysis.

### Statistical analysis

For serum parameters, western blotting and IHC analysis, each piglet was used as a statistical unit. Experimental data are presented as means ± SEs. Statistical significance was analyzed by one way ANOVA with a general linear model, followed by Duncan’s multiple range tests using SAS software (SAS Institute, Cary, NC, USA). Significance was determined at *P* < 0.05.

## Results

### Serum antioxidant characteristics, inflammatory factors, and rectal temperature

**Fig. 1:**
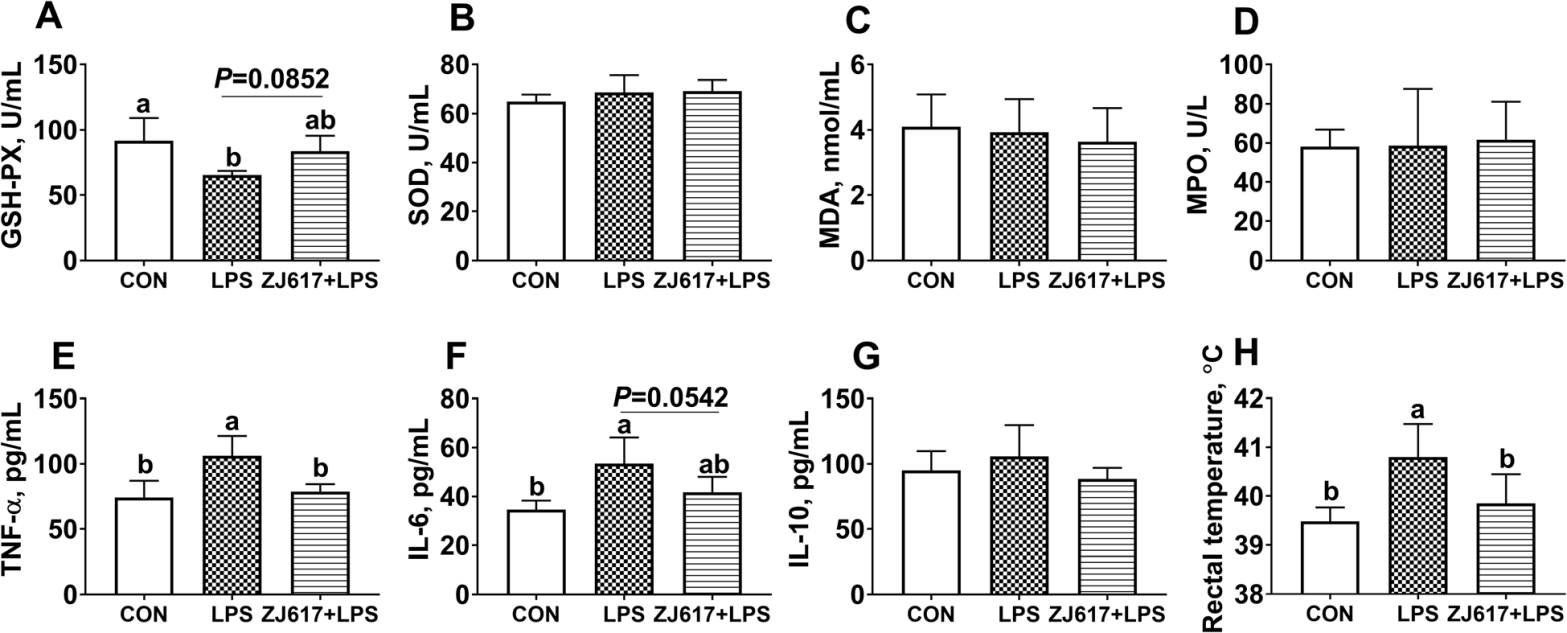
Serum biochemical parameters in piglets of the three treatment groups. **A**-**H**: GSH-Px, SOD, MDA, MPO, TNF- *α,* IL-6, IL-10, respectively. CON, control piglets were given PBS orally. LPS group, received intraperitoneal injection (i.p.) of LPS (25 μg/kg body weight); ZJ617 + LPS, piglets orally inoculated with ZJ617 (1 × 10^10^ CFU/d) for 2 weeks before i.p. injection of LPS. Values are shown as means ± SEs, *n* = 6. Labeled means without a common letter differ, *P* < 0.05. GSH-Px, glutathione peroxidase; LDH, lactate dehydrogenase; MDA, malonic dialdehyde; MPO, myeloperoxidase; IL-6, interleukin-6; IL-10, interleukin-10; SOD, superoxide dismutase; TNF-*α*, tumor necrosis factor-α

LPS stimulation significantly decreased serum GSH-Px concentrations in piglets compared with the CON group. Administration of ZJ617 showed a trend for higher serum GSH-Px concentrations compared with the LPS group (*P* = 0.0852, Fig. 1A). No significant differences were observed in SOD, MDA, and MPO, across the three groups (Fig. 1B-D).

Serum TNF-α and IL-6 levels were significantly higher in the LPS group than in the CON group (*P* < 0.05) (Fig. 1E, F). ZJ617 pretreatment significantly lowered TNF-α concentrations (*P* < 0.05) (Fig. 1E) and showed a trend for decreasing IL-6 concentrations (*P* = 0.0542) compared with the LPS group (Fig. 1F). There were no significant differences in serum IL-10 levels among the three groups (Fig. 1G). The rectal temperature of the piglets in the LPS group was significantly higher than in piglets in the CON group, whereas ZJ617 pretreatment significantly decreased rectal temperatures of piglets in the ZJ617 + LPS group (*P* < 0.05, Fig. 1H).

### Serum characteristics as indicators of intestinal permeability and liver function

**Fig. 2:**
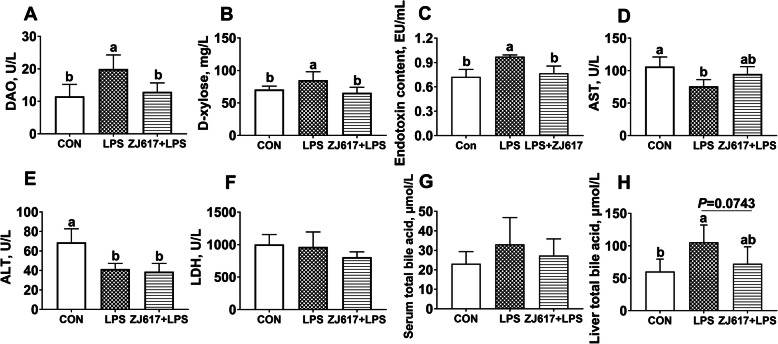
Serum biochemical parameters in piglets of three treatment groups. **A**-**H**: DAO, D-xylose, endotoxin, AST, ALT, LDH, serum total bile acid, liver total bile acid. CON, control piglets orally inoculated with PBS; LPS, piglets received intraperitoneal injection (i.p.) of LPS (25 μg/kg body weight); ZJ617 + LPS, piglets orally inoculated with ZJ617 (1 × 10^10^ CFU/d) for 2 weeks before i.p. injection of LPS. Values are shown as means ± SEs, *n* = 6. Labeled means without a common letter differ, *P* < 0.05. DAO, diamineoxidase; AST, aspartate aminotransferase; ALT, alanine aminotransferase; LDH, lactate dehydrogenase; PBS, phosphate-buffered saline

The LPS challenge significantly increased serum DAO activities and D-xylose levels compared with the control group (*P* < 0.05) (Fig. 2A, B). However, compared with the LPS group, ZJ617 pretreatment significantly decreased serum DAO and D-xylose levels (*P* < 0.05) (Fig. 2A, B). LPS injection alone significantly increased serum LPS levels in the LPS group (*P* < 0.05), whereas ZJ617 pretreatment significantly attenuated the rise of serum LPS levels in the ZJ617 + LPS group compared with LPS group (*P* < 0.05, Fig. 2C).

Serum AST and ALT levels were significantly lower in the LPS group than in the CON group, which implied that the use of LPS caused liver dysfunction. However, there was no significant difference in ALT and AST levels between the ZJ617 + LPS and LPS treatment groups (Fig. 2D, E). There was no significant difference in LDH levels among three groups (Fig. 2F). We assessed the level of TBA in the serum and liver and found that there was no significant difference in serum TBA levels across the three groups (Fig. 2G). However, hepatic TBA levels were significantly elevated after LPS stimulation compared with the CON group (*P* < 0.05) and ZJ617 pretreatment showed a tendency to prevent the increase in TBA in the ZJ617 + LPS group compared with the LPS group (*P* = 0.0743, Fig. 2H).

### Expression of intestinal inflammatory signaling pathways elements

**Fig. 3:**
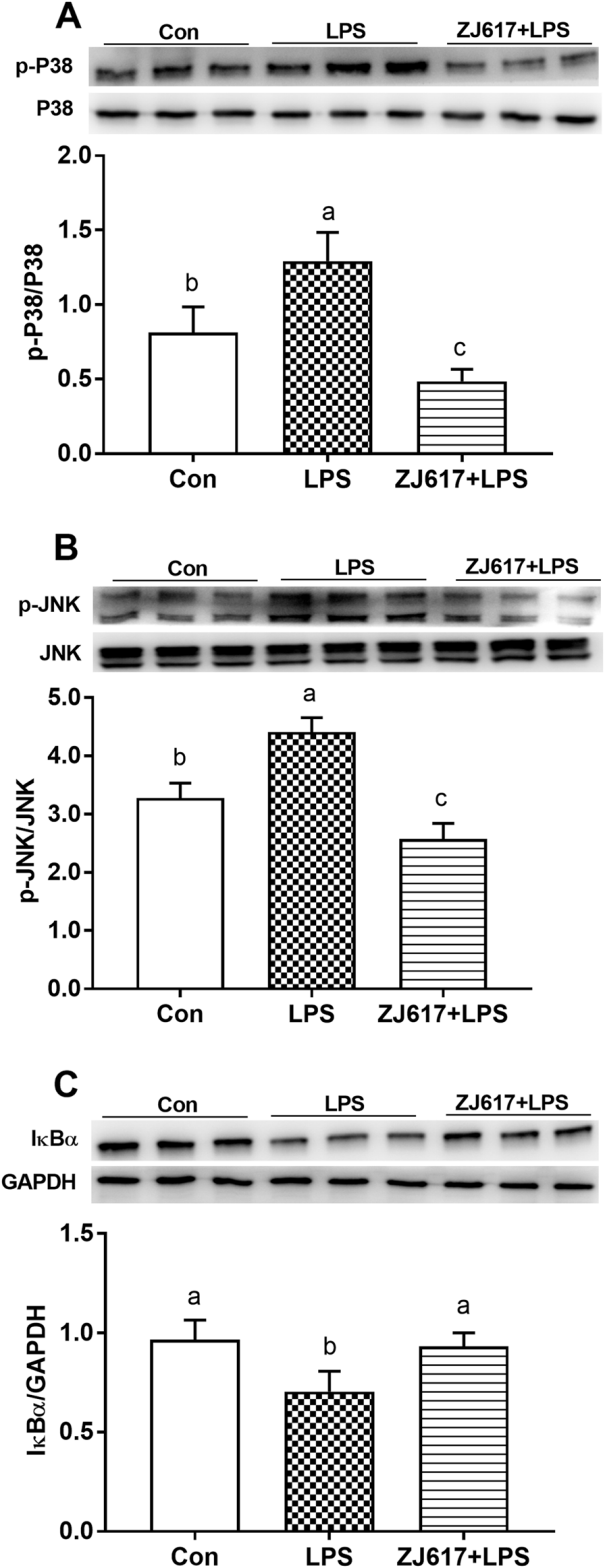
Western blotting evaluation of the expression of cell signaling molecules in the ilea of piglets of the three treatment groups. **A**-**C**: p-p38/p38, p-JNK, IκBα, respectively. CON, control piglets orally inoculated with PBS; LPS, piglets received intraperitoneal injection (i.p.) of LPS (25 μg/kg body weight); ZJ617 + LPS, piglets orally inoculated with ZJ617 (1 × 10^10^ CFU/d) for 2 weeks before i.p. injection of LPS. The protein bands were quantified by densitometry analysis and normalized to the level of GAPDH. Values are shown as means ± SEs, *n* = 6. Labeled means without a common letter differ, *P* < 0.05. IκBα, nuclear factor-kappa B inhibitor alpha; p-, phospho-; p38, mitogen-activated protein kinase (MAPK) p38; JNK, c-Jun N-terminal kinase

**Fig. 4:**
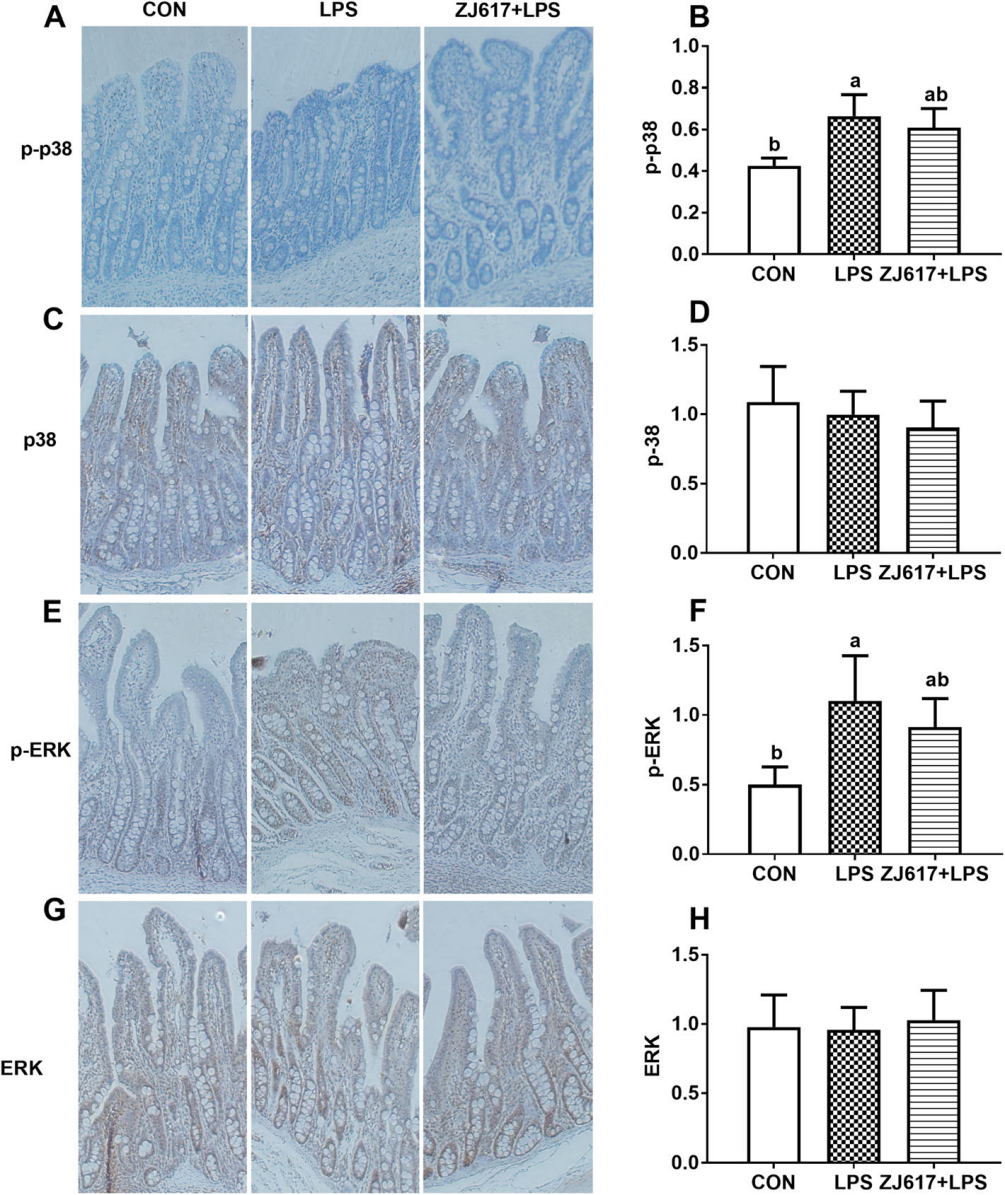
Immunohistochemistry analysis of the expression of cell signaling molecules in the ilea piglets of the three treatment groups. **A**-**D**: Staining indicates p-p38, p38, p-ERK, and ERK positive cells, respectively. CON, control piglets orally inoculated with PBS; LPS, piglets received intraperitoneal injection (i.p.) of LPS (25 μg/kg body weight); ZJ617 + LPS, piglets orally inoculated with ZJ617 (1 × 10^10^ CFU/d) for 2 weeks before i.p. injection of LPS. Ratios of positively stained cells to total cells are shown as means ± SEs, *n* = 6. Labeled means without a common letter differ, *P* < 0.05. p-, phospho-; p38, mitogen-activated protein kinase (MAPK) p38; ERK, extracellular signal-regulated kinase

Piglets in the LPS group had significantly higher expression of phosphorylated p38 (p-p38 MAPK) (Fig. 3A) and p-JNK (Fig. 3B) levels but decreased IκBα (Fig. 3C) levels than the CON group (*P* < 0.05). Compared to the LPS group alone, ZJ617 pretreatment significantly reduced the intestinal p-p38 MAPK and p-JNK levels, whereas it increased IκBα levels (*P* < 0.05, Fig. 3). Immunohistochemical analysis demonstrated that the expression of p-p38 MAPK (Fig. 4A, B) and phosphorylated-ERK (p-ERK) (Fig. 4E, F) in the LPS group was significantly elevated compared to the CON group (*P* < 0.05). The ZJ617 + LPS group showed no significant differences in the expression of p-p38 MAPK (Fig. 4A, B) and p-ERK (Fig. 4E, F) compared with the LPS group (*P* > 0.05). There were no significant differences in p-p38 MAPK (Fig. 4C, D) and p-ERK (Fig. 4G, H) expression levels among the three treatment groups (*P* > 0.05).

### Expression of intestinal tight junction proteins

**Fig. 5:**
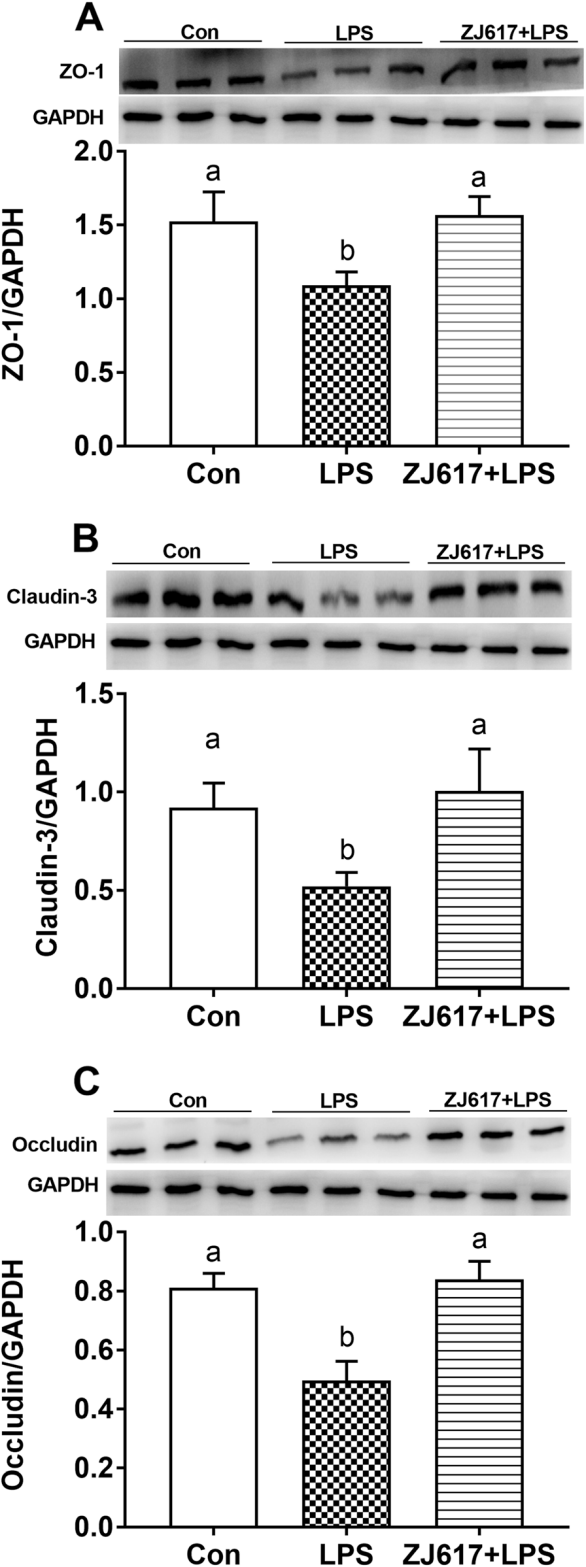
Western blotting evaluation of the expression of ilea tight junction proteins in piglets of the three treatment groups. **A**-**C**: ZO-1, Cluaudin-3, Occludin, respectively. CON, control piglets orally inoculated with PBS; LPS, piglets received intraperitoneal injection (i.p.) of LPS (25 μg/kg body weight); ZJ617 + LPS, piglets orally inoculated with ZJ617 (1 × 10^10^ CFU/d) for 2 weeks before i.p. injection of LPS. ZO-1, zonula occludens-1. The protein bands were quantified by densitometry analysis and normalized to the level of GAPDH. Values are shown as means ± SEs, *n* = 6. Labeled means without a common letter differ, *P* < 0.05

**Fig. 6:**
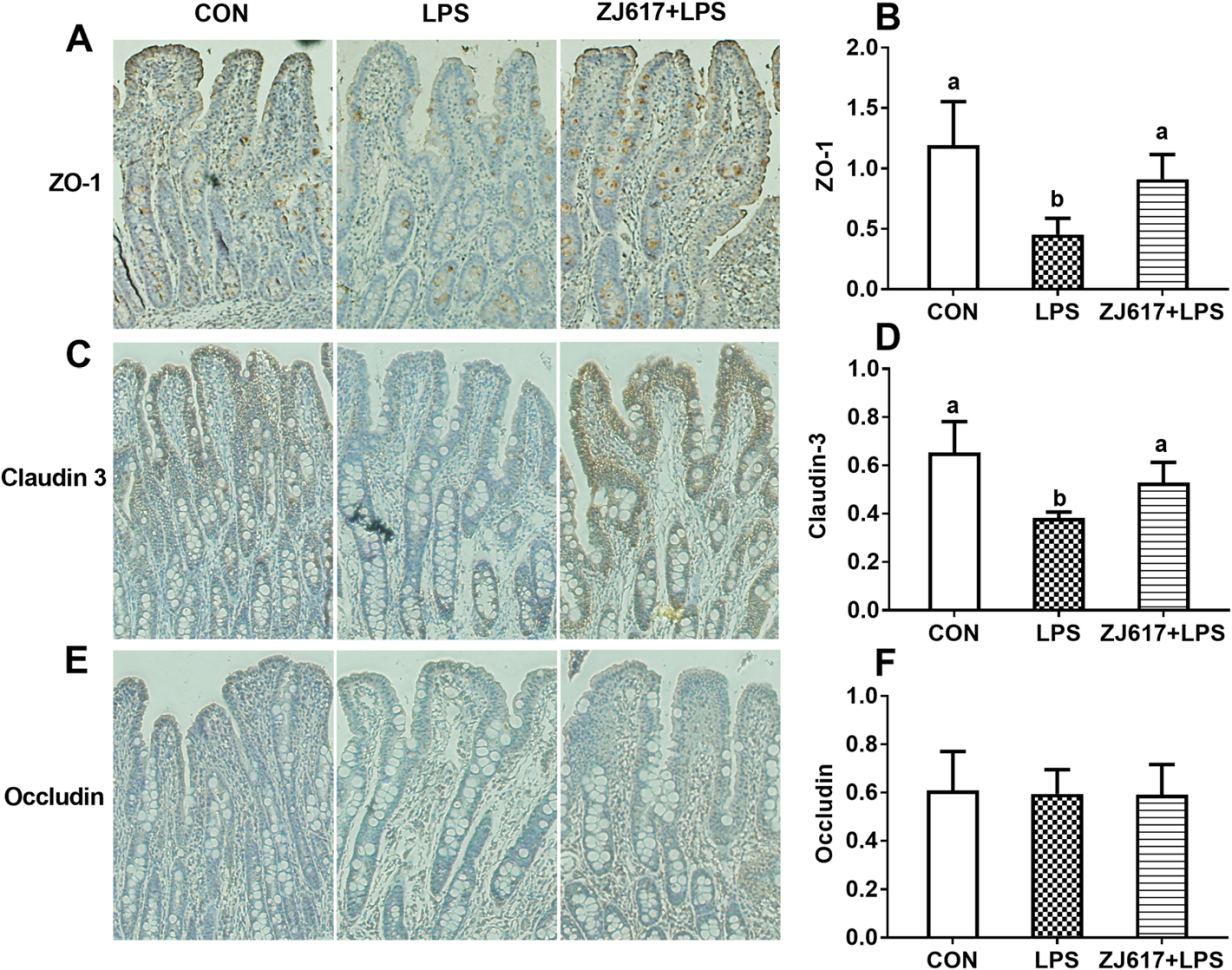
Immunohistochemistry analysis of the expression of ilea tight junction proteins in piglets of three groups. **A**-**F**: Staining indicates ZO-1, claudin-3, and occludin positive cells, respectively. CON, control piglets orally inoculated with PBS; LPS, piglets received intraperitoneal injection (i.p.) of LPS (25 μg/kg body weight); ZJ617 + LPS, piglets orally inoculated with ZJ617 (1 × 10^10^ CFU/d) for 2 weeks before i.p. injection of LPS. ZO-1, zonula occludens-1. Ratios of positively stained cells to total cells are shown as means ± SEs, *n* = 6. Labeled means without a common letter differ, *P* < 0.05

Compared with the CON group, LPS stimulation significantly decreased intestinal ZO-1 (Fig. 5A) claudin-3 (Fig. 5B), and occludin (Fig. 5C) protein expression in LPS group piglets (*P* < 0.05). ZJ617 pretreatment significantly increased ZO-1 (Fig. 5A), claudin-3 (Fig. 5B), and occludin (Fig. 5C) protein expression in the ZJ617 + LPS group compared with the LPS group (*P* < 0.05). Immunohistochemical analysis further confirmed that LPS stimulation significantly lowered ZO-1 (Fig. 6A, B) and claudin-3 (Fig. 6C, D) protein expression compared with the CON group (*P* < 0.05), whereas ZJ617 pretreatment significantly increased ZO-1 (Fig. 6A, B) and claudin-3 (Fig. 6C, D) protein expression compared with the LPS group (*P* < 0.05). However, immunohistochemical analysis showed there was no significant difference in occludin expression among three groups (Fig. 6E, F). LPS stimulation significantly decreased villus height compared with the CON group (*P* < 0.05, Supplementary Fig. S1); however, ZJ617 pretreatment significantly increased villus height compared with the LPS group (*P* < 0.05, Supplementary Fig. S1). There were no significant differences in crypt depth and the ratio of villus height to crypt depth (*P* > 0.05, Supplementary Fig. S1).

### Intestinal autophagy and apoptosis signaling protein expression

**Fig. 7:**
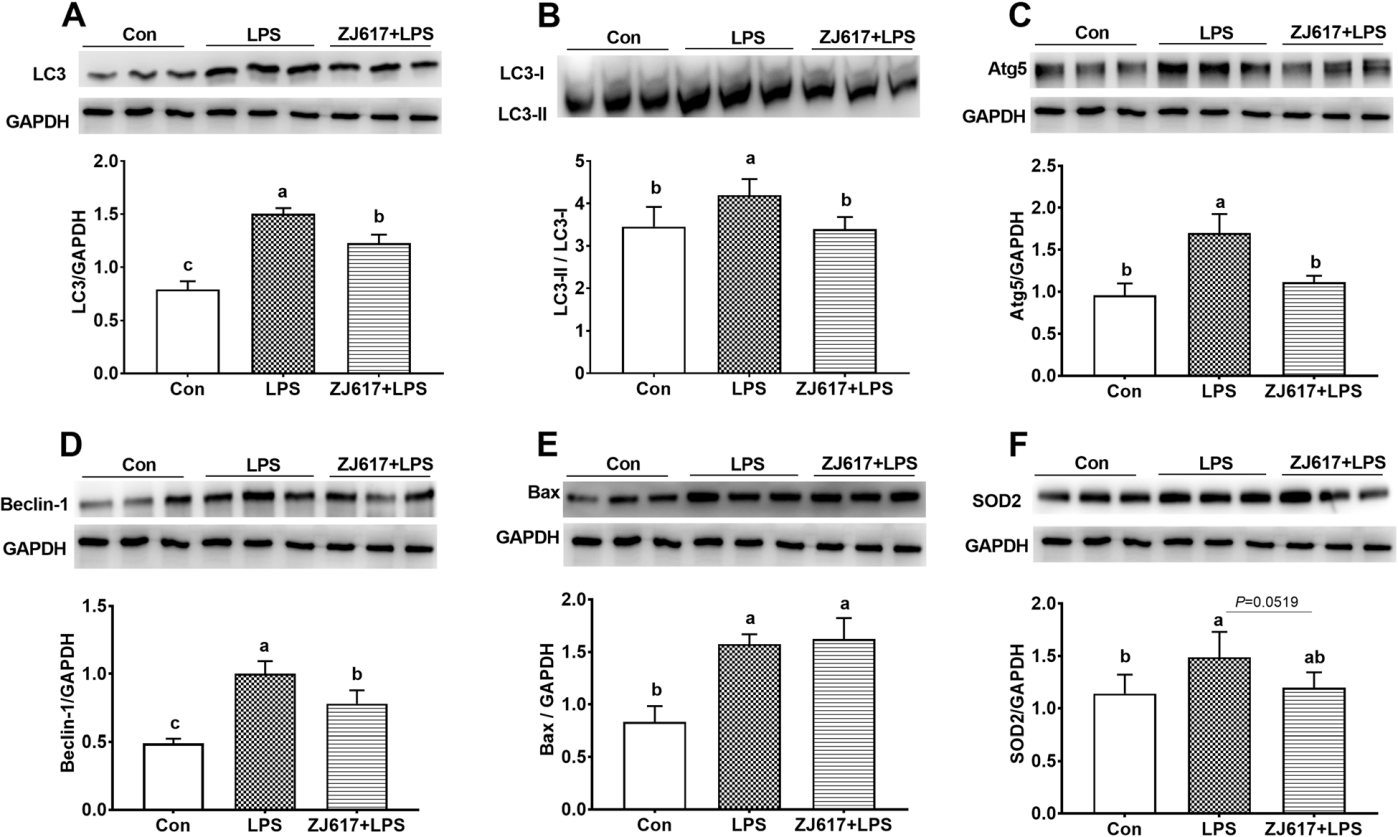
Western blotting evaluation of the expression of autophagy signaling proteins in ilea tissues of piglets of the three treatment groups. **A**-**F**: LC3, LC3-II/LC3-I, Atg5, Beclin-1, Bax, SOD2, respectively. CON, control piglets orally inoculated with PBS; LPS, piglets received intraperitoneal injection (i.p.) of LPS (25 μg/kg body weight); ZJ617 + LPS, piglets orally inoculated with ZJ617 (1 × 10^10^ CFU/d) for 2 weeks before i.p. injection of LPS. The protein bands were quantified by densitometry analysis and normalized to the level of GAPDH. Values are shown as means ± SEs, *n* = 6. Labeled means without a common letter differ, *P* < 0.05. Atg5, autophagy-related protein 5; Bax, B-cell lymphoma-2 associated X protein; LC3, microtubule-associated protein 1 light chain 3; SOD2, superoxide dismutase 2

Western blotting analysis demonstrated that the LPS group had significantly higher light chain 3 (LC3), ratio of LC3-II to LC3-I (LC3-II/LC3-I), autophagy-related protein 5 (Atg5), and Beclin-1 expression than the CON groups (*P* < 0.05, Fig. 7A-D). Compared with the LPS group, the administration of ZJ617 significantly decreased LC3, LC3-II/LC3-I, Atg5, and Beclin-1 expression (*P* < 0.05, Fig. 7A-D). In the apoptosis pathway, LPS stimulation led to a significantly higher levels of Bax compared with the CON group (*P* < 0.05, Fig. 7E). However, ZJ617 administration failed to decrease intestinal Bax expression levels in the ZJ617 + LPS group compared to the LPS-treated group (*P* > 0.05, Fig. 7E). With regard to antioxidant capacity, LPS stimulation led to a significantly higher expression of superoxide dismutase 2 (SOD2) compared with the CON group (*P* < 0.05, Fig. 7F). ZJ617 administration showed a trend to decrease SOD2 expression levels in ZJ617 + LPS group compared with the LPS group (*P* = 0.0519, Fig. 7F).

### Hepatic autophagy and apoptosis signaling expression

**Fig. 8:**
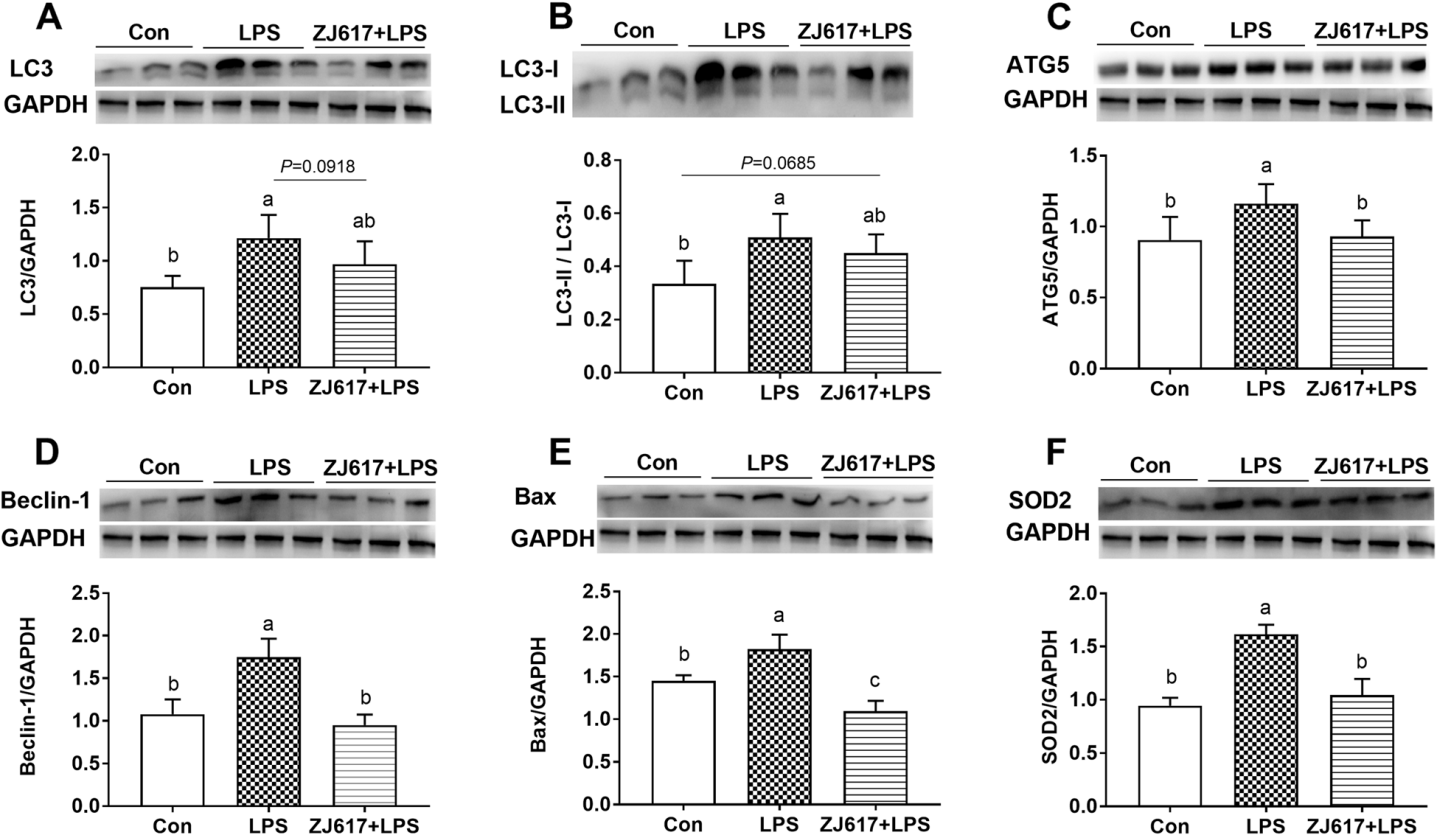
Western blotting evaluation of the expression of hepatic autophagy and apoptosis signaling proteins in the ilea tissues of piglets of the three treatment groups. A-F: LC3, LC3-II/LC3-I, Atg5, Beclin-1, Bax, SOD2, respectively. CON, control piglets orally inoculated with PBS; LPS, piglets received intraperitoneal injection (i.p.) of LPS (25 μg/kg body weight); ZJ617 + LPS, piglets orally inoculated with ZJ617 (1 × 10^10^ CFU/d) for 2 weeks before i.p. injection of LPS. The protein bands were quantified by densitometry analysis and normalized to the level of GAPDH. Values are shown as means ± SEs, n = 6. Labeled means without a common letter differ, *P* < 0.05. Atg5, autophagy-related protein 5; Bax, B-cell lymphoma-2 associated X protein; LC3-II, microtubule-associated protein 1 light chain 3; SOD2, superoxide dismutase protein 2

We used western blotting to evaluate the expression of several key proteins involved in the autophagy and apoptosis signaling to investigate whether ZJ617 exerted any effects on these pathways. The expression of LC3, LC3-II/LC3-I, Atg5, and Beclin-1 were significantly elevated in the liver after LPS stimulation compared with the CON group (*P* < 0.05, Fig. 8A-D). The administration of ZJ617 significantly lowered the expression of Atg5 (Fig. 8C) and Beclin-1 (Fig. 8D) (*P* < 0.05) and showed a trend to decrease LC3 (*P* = 0.0918, Fig. 8A). However, there was no significant difference in the LC3-II/LC3-I ratio between the LPS and ZJ617 + LPS groups (*P* > 0.05, Fig. 8B). In the apoptosis pathway, LPS stimulation led to a significantly higher expression of Bax compared with the CON group (*P* < 0.05, Fig. 8E). ZJ617 administration significantly decreased Bax expression levels in the ZJ617 + LPS group compared with the LPS and CON groups (*P* < 0.05, Fig. 8E). For antioxidant capacity analysis, LPS stimulation led to a significantly higher expression of SOD2 compared with the CON group (*P* < 0.05, Fig. 8F). ZJ617 administration significantly decreased SOD2 expression levels in the ZJ617 + LPS group compared with the LPS-treated group (*P* < 0.05, Fig. 8F).

### Metabolomics of intestinal contents

**Fig. 9:**
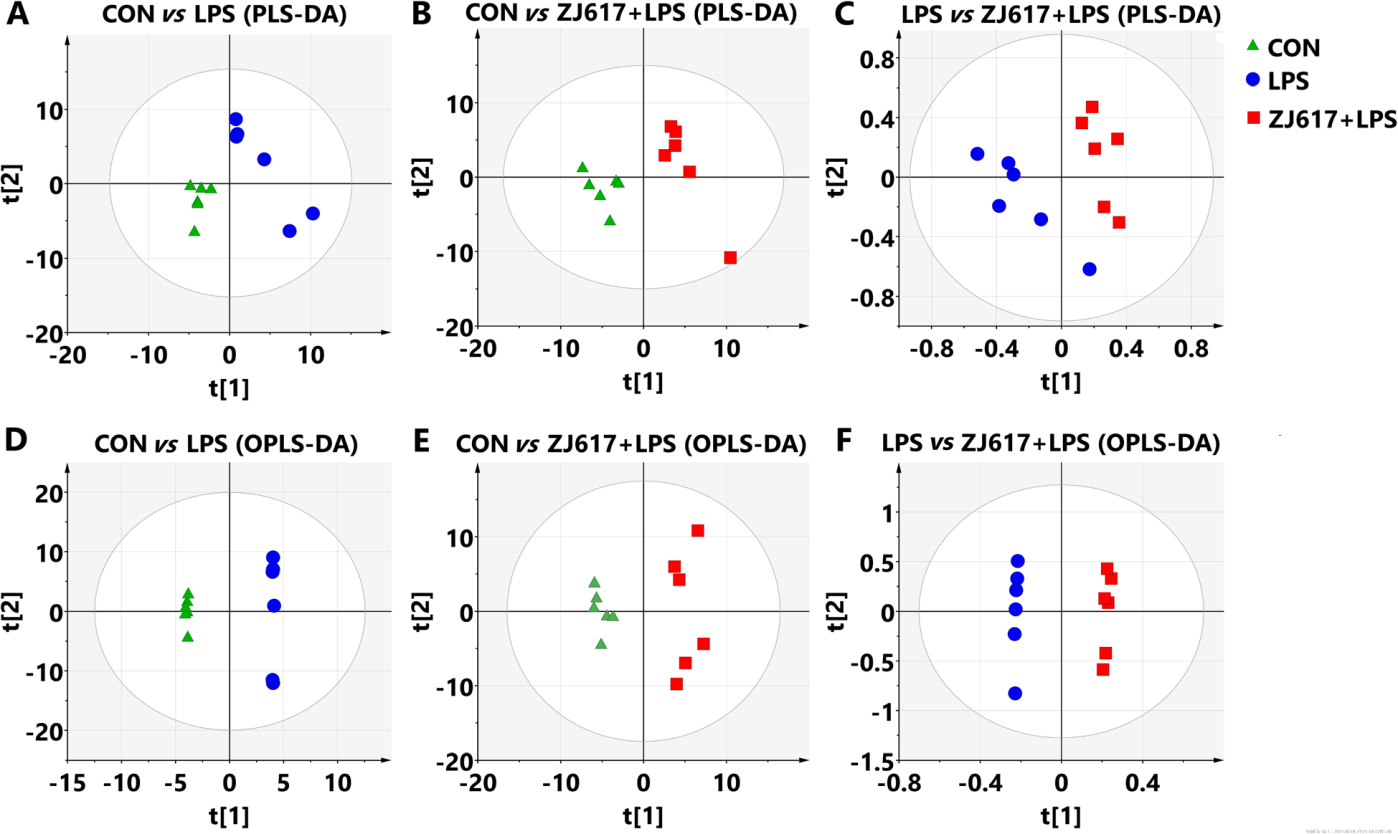
Score plot from PLS-DA (**A**–**C**) and OPLS-DA (**D**–**F**) of metabolite profiles of intestinal contents in piglets between each of the 2 groups from CON, LPS, and ZJ617 + LPS groups. CON, control piglets orally inoculated with PBS (▲); LPS, piglets following intraperitoneal injection (i.p.) with LPS (25 μg/kg body weight) (●); ZJ617 + LPS, piglets orally inoculated with ZJ617 (1 × 10^10^ CFU/d) for 2 weeks before i.p. injection of LPS (■). Each data point represents a function of the entire spectral profile of each subject (*n* = 6). OPLS-DA, orthogonal projections to latent structures-discriminant analysis; PLS-DA, partial least squares-discriminant analysis

Next, we analyzed LPS-induced changes in the metabolites of intestinal contents. A total of 914 metabolite peaks were identified with the help of LECO/Fiehn Metabolomic Library. In order to further identify the classification of sample categories, PLS-DA and OPLS-DA approaches were utilized to establish the relationship between metabolite expression and the sample category. We observed that there was a clear distinction between the CON and the LPS groups (Fig. 9A, D), the CON and ZJ617 + LPS groups (Fig. 9B, E), and the LPS and ZJ617 + LPS groups (Fig. 9C, F) based on the PLS-DA and OPLS-DA models, suggesting that each sample in the three groups was clearly separated.

We used fold change (FC) to evaluate the quantity of metabolites in the CON, LPS, and ZJ617 + LPS groups. LPS treatment induced a significant increase in 4 metabolites in the intestinal contents: dihydrocholesterol (FC = 0.20), 1-deoxyerythritol (FC = 0.21), erythronic acid (FC = 0.3), and glutaric acid (FC = 0.42) (*P* < 0.05, Table 1). Compared to the CON group, preventative ZJ617 administration before LPS injection resulted in 5 significantly increased metabolites in the intestinal contents (Table 2): glutamine 4TMS minor1 (FC = 0.09), phenylacetic acid (FC = 0.23), 2-monopalmitin (FC = 0.21), 2-deoxytetronic acid NIST (FC = 0.21), and erythronic acid (FC = 0.31) (*P* < 0.05). Compared to the LPS group, pretreatment with ZJ617 before LPS injection also led to 5 significantly increased metabolites in the intestinal contents: capric acid (FC = 0.03), isoleucine 1TMS (FC = 0.02), glycerol-1-phosphate byproduct (FC = 0.18), linoleic acid (FC = 0.09), and alanine-alanine (FC = 0.03) (*P* < 0.05, Table 3).

**Table 1:** List of significantly altered metabolites in the intestine of piglets between CON and LPS groups

Metabolite	Similarity	R.T.	Mass	VIP	*P* value	FC
1-Mono-olein	727.11	22.65	399	1.80	0.0394	2.52
1-Deoxyerythritol	764.13	8.13	91	1.70	0.0088	0.21
3,4-Dihydroxyphenylacetic acid	876.07	15.47	147	1.55	0.0454	2.74
Dihydrocholesterol	914.89	6.33	130	1.62	0.0154	0.20
Erythritol	811.63	6.39	241	1.65	0.0338	5.34
Erythronic acid	616.93	16.51	174	1.80	0.0296	0.30
Glutaric acid	731.94	10.85	174	1.51	0.0129	0.42
Isoleucine minor	911.98	18.16	95	1.92	0.0253	1.98
Methionine	874.30	19.5	91	1.74	0.0272	3.89
Sophorose minor	847.31	20.2	204	1.68	0.0346	3.01

*CON* control piglets orally inoculated with PBS, *LPS* piglets received intraperitoneal injection (i.p.) with lipopolysaccharides (25 μg/kg body weight)

Metabolites with VIP > 1.5 are shown in the Table. FC, fold-change of the peak intensity for the CON group against the LPS group (*n* = 6); *P* values were calculated according to Student’s *t* test

*RT*, retention time; *VIP*, variable importance in projection

**Table 2:** List of significantly altered metabolites in the intestine of piglets between CON and ZJ617 + LPS groups

Metabolite	Similarity	R.T.	Mass	VIP	*P* value	FC
Glutamine 4TMS minor	854.9641	11.57	129	2.25	0.0007	0.09
Phenylacetic acid	939.3611	23.96	204	2.14	0.0007	0.23
Alanine	839.9175	11.35	115	2.07	0.0049	2.60
Glycine	730.6577	5.77	147	2.06	0.0025	1.75
Agmatine	741.8409	20.1	131	1.95	0.0044	1.38
2-Monopalmitin	649.5071	22.29	361	1.80	0.0408	0.21
2-Deoxytetronic acid NIST	870.2739	15.66	147	1.78	0.0120	0.21
Erythronic acid	616.9285	16.51	174	1.71	0.0360	0.31
Capric acid	901.6143	22	204	1.70	0.0567	0.04
z-C08 FAME internal standard	611.1466	4.48	207	1.63	0.0426	1.34
z-Hexose perTMS	985.0225	4.82	147	1.60	0.0522	0.82
Methionine sulfoxide minor	602.5514	5.26	151	1.58	0.0384	1.30
Allantoic acid (dehydrated) 3TMS minor	650.7138	21	91	1.57	0.0279	3.07

*CON* control piglets orally inoculated with PBS; ZJ617 + LPS, piglets orally inoculated with ZJ617 (1 × 10^10^ CFU/d) for 2 weeks before i.p. injection of lipopolysaccharides (25 μg/kg body weight). Metabolites with VIP > 1.5 are shown in the Table. FC, fold-change of the peak intensity for the CON group against the LPS + ZJ617 group (*n* = 6); *P* values were calculated according to Student’s *t* test

*ZJ617*, *Lactobacillus reuteri* ZJ617; *RT*, retention time; *VIP*, variable importance in projection

**Table 3:** List of significantly altered metabolites in the intestine of piglets between LPS and ZJ617 + LPS groups

Metabolite	Similarity	R.T.	Mass	VIP	*P* value	FC
Capric acid	901.6143	22	204	2.23	0.0299	0.03
Isoleucine 1TMS	800.1796	15.4	319	2.19	0.0327	0.02
Glycerol-1-phosphate byproduct	627.0337	21.49	361	1.94	0.0396	0.18
Linoleic acid	941.0033	23.04	204	1.87	0.0415	0.09
Alanine-alanine	812.2268	21.87	204	1.60	0.0289	0.03

*LPS* piglets received intraperitoneal injection (i.p.) of lipopolysaccharides (25 μg/kg body weight); ZJ617 + LPS, piglets orally inoculated with ZJ617 (1 × 10^10^ CFU/d) for 2 weeks before i.p. injection of lipopolysaccharides (25 μg/kg body weight)

ZJ617, *Lactobacillus reuteri* ZJ617; FC, fold-change of the peak intensity for the LPS group against the LPS + ZJ617 group (*n* = 6)

*P* values were calculated according to Student’s *t* test. Metabolites with VIP > 1.5 are shown in the table; *RT*, retention time; *VIP*, variable importance in projection

Based on the variations in metabolite concentrations mentioned above, significant changes were found in 8 metabolic pathways by KEGG pathway analysis. Compared to the CON group and LPS groups, four metabolite pathways (valine, leucine and isoleucine biosynthesis; aminoacyl-tRNA biosynthesis; glycine, serine and threonine metabolism; and arginine biosynthesis) were altered after the administration of ZJ617 (Table 4). LPS stimulation could alter alanine, aspartate, and glutamate metabolism independently of ZJ617 pretreatment (Table 4). LPS also altered glyoxylate and dicarboxylate metabolism in the LPS group compared with the CON group. The intervention with ZJ617 influenced phenylalanine metabolism and biosynthesis of unsaturated fatty acids compared with CON and LPS groups, respectively (Table 4).

**Table 4:** Altered metabolic pathways identified based on significant changes in concentrations of metabolites in the piglet intestine in 2 group comparisons

Group comparison	Altered metabolic pathway
CON vs LPS	Alanine, aspartate, and glutamate metabolism Glyoxylate and dicarboxylate metabolism
CON vs ZJ617 + LPS	Valine, leucine and isoleucine biosynthesis Aminoacyl-tRNA biosynthesis Glycine, serine and threonine metabolism Biosynthesis of unsaturated fatty acids Arginine biosynthesis
LPS vs ZJ617 + LPS	Aminoacyl-tRNA biosynthesis Valine, leucine and isoleucine biosynthesis Alanine, aspartate and glutamate metabolism Phenylalanine metabolism Glycine, serine and threonine metabolism Arginine biosynthesis

*CON* control piglets orally inoculated with PBS; *LPS* piglets i.p. injected with LPS (25 μg/kg body weight); ZJ617 + LPS, piglets orally inoculated with ZJ617 (1 × 10^10^ CFU/d) for 2 weeks before intraperitoneal injection (i.p.) of LPS. *ZJ617*, *Lactobacillus reuteri* ZJ617

## Discussion

Previous studies have shown that external stimuli, including the LPS component of gram-negative bacteria, could induce intestinal barrier dysfunction through various mechanisms [13, 47, 48], these studies confirmed that LPS disrupts intestinal barrier by down-regulating the expression of TJ proteins. Although, there are many TJ proteins present in the intestinal tissue, ZO-1, occludin, and claudin-3 are most often selected as the representative TJ proteins. The mRNA and protein expression of ZO-1, occludin, and claudin-1 were all down-regulated by LPS in a Caco-2 cell monolayer model [49]. Likewise, LPS induced the impairment of barrier integrity through a decrease in the synthesis of claudin-3 and 4 in IPEC-J2 cells [50]. As we hypothesized, ZJ617 could restore TJ proteins expression down-regulated by LPS. The protective effect of *L. reuteri* on the intestinal barrier has also been verified in other studies. The transcripts of TJ protein ZO-1, claudin-1 and occludin increased in the mucosa of the jejunum and ileum in weaned pigs treated with *L. reuteri* LR1 for 2 weeks [5]. *L. reuteri* LR1 also increased transcript abundance and protein contents of ZO-1 and occludin in ETEC K88-infected IPEC-1 cells [5]. As shown in these studies, *L. reuteri* strains could play an important role in improving the expression of TJ proteins.

We found LPS stimulation alone led to higher serum LPS levels and consistently lower expression of TJ proteins compared with other treatments. Previous studies have shown that increased intestinal barrier permeability correlated with serum LPS, indicating that plasma levels of LPS, zonulin, and FABP2 (biomarkers of the gastrointestinal epithelium TJ barrier integrity) were consistently significantly elevated [51]. Lowering of TJ protein levels weakens the connection between intestinal epithelial cells, leading to the decline of barrier function, which allows easier access to the external substances to translocate into the body. The increase in D-xylose and DAO in the serum was also suggestive of damage to the intestinal barrier function in the LPS group. *L. paracasei* Jlus66 could attenuate oxidative stress by decreasing MDA levels and enhancing SOD and GSH-Px levels [52]. Although, there was no difference in SOD or MDA expression among the three groups, we found that serum GSH-Px concentrations in the ZJ617 + LPS group were higher than that in the LPS group, suggesting that ZJ617 interfered with oxidative stress.

Higher serum LPS levels may easily result in intestinal and systemic inflammatory responses. In our study, the rise of rectal temperature acted as an external manifestation of systemic inflammation. The activation of various intracellular signaling pathways indicates that inflammation is triggered and the immune system is activated. As an inflammatory agent, LPS binds to the membrane receptor TLR4 and subsequently triggers the TLR/MAPK/NF-κB signaling pathways to secret an abundance of inflammatory cytokines that ultimately leads to tissue damage [53]. Our previous results indicated that the supernatant of ZJ617 could reduce TLR/MAPK/NF-κB signaling in the liver of a murine model [20]. In this study, western blotting and immunohistochemical analysis demonstrated that ZJ617 could interfere with inflammatory pathways by decreasing the phosphorylation of p38 MAPK, JNK, ERK, and increasing IκBα levels. Elevated IκBα reduces the degradation of IκBα, which leads to the reduced activation of the downstream NF-κB pathway. NF-κB is an important regulator of cellular gene transcription and is closely associated with the production of many cytokines [54]. TNF-α, IL-6 and IL-10 are cytokines produced during the immune reaction; the concentration of these cytokines was notably elevated in serum after LPS stimulation. We found that the administration of ZJ617 reduced the increase in cytokine levels, which was consistent with previous results in a murine model [43]. Several cytokines including TNF-α and interleukins such as IL-6, IL-8, and IL-10 are involved in the development of inflammation. The activation of TLR4 signaling increases proinflammatory cytokine production including that of TNF [55], which in turn induces the release of other proinflammatory cytokines (e.g., IL-1β, and IL-6) [56]. Thus, the production of cytokines is down-regulated when the signaling pathway is inhibited. The decreased concentration of signaling molecules and cytokines demonstrated that ZJ617 suppressed inflammatory signaling pathways, and thus reduced the production of cytokines.

Substantial evidence has revealed that LPS stimulation triggers the activation of autophagy, inflammation, and apoptosis signaling pathways [49, 57, 58] and increased oxidative stress [59]. Compared with the CON group, the higher expression levels of LC3, Atg5, Beclin-1, Bax, and SOD2 in the intestine and liver of the LPS group indicated that LPS challenge elevated the autophagy, apoptosis, and oxidative stress levels. Several animal studies have shown that LPS-induced autophagy has a protective effect on tissue injury. Compared with mice carrying a control vector, mice developed less severe liver injury when the transcription factor EB (TFEB) was overexpressed in the liver and TFEB is required for autophagy [60]. Boosting autophagy quenched intestinal inflammation and oxidative stress injury [61]. Furthermore, intestinal inflammation was ameliorated by promoting autophagy in murine colitis models [62]. These results showed that the progression of inflammatory activity was often accompanied by the induction of autophagy. In addition, there have been numerous studies investigating the relationship between the TJ barrier and autophagy. The increase of the reduction and reorganization of TJ proteins in sham-burned mice was the result of inducing autophagy with rapamycin [63]. However, these findings showed that autophagy could enhance the TJ barrier by inhibiting beclin-1 function, which regulated intestinal TJ barrier function via the endocytosis of occludin [64]. In this study, lower levels of autophagy, apoptosis, oxidative stress, and higher TJ proteins expression in ZJ617 + LPS group indicated that pretreatment with ZJ617 could alleviate the degree of tissue damage, both in the liver and in the intestine of piglets stimulated by LPS. Accordingly, pretreatment with ZJ617 restored the intestinal villus height and improved the structure of villi morphology.

Bile acids are synthesized from cholesterol in the liver and their secretion by hepatocytes will generate bile flow. Normally, bile is secreted into the intestine and once the liver is damaged, bile acid secretion will be blocked. TBA has been considered biomarkers of liver injury for decades. In our study, the TBA levels in the liver and serum in the LPS group was significantly higher than in the ZJ617 + LPS group, indicating that ZJ617 effectively reduced the liver injury induced by LPS. LGG has also been reported to significantly reduced bile acid levels by hepatic FXR activation and upregulation of the bile salt export pump [63]. Thus, *lactobacillus* plays an important role in alleviating liver injury, although the mechanism involved warrants further study.

Dihydrocholesterol is a product of the cholesterol synthesis pathway and may be related to the formation of cholic acid and hormones. Previous studies revealed that LPS decreased hepatic cholesteryl ester transfer protein (CETP) expression, which plays an important role in the retrograde transport system of cholesterol [65]. This may explain the decrease of dihydrocholesterol in intestinal contents after LPS injection, although the underlying mechanism still needs to be explored.

We found that a total of 8 metabolic pathways were significantly altered following exposure to ZJ617, which suggesting that ZJ617 have potential to regulate intestinal metabolism in piglets. Numerous animal studies have shown that *Lactobacillus* species are involved in regulating the host’s metabolism. *L. gasseri* SBT2055 modulated fat absorption and excretion in lean rats [66]; expression of glycogen synthesis related genes (GSK-3β) and lipogenic-related genes (FAS and SREBP-1c) were reduced after *L. acidophilus* KLDS1.1003 and KLDS1.0901 treatment, while the insulin resistance associated gene Akt was up-regulated [36]. Metabolomic analysis showed that ZJ617 mainly affected the biosynthesis of amino acids: valine, leucine, and isoleucine biosynthesis; aminoacyl-tRNA biosynthesis; glycine, serine, and threonine metabolism; and arginine biosynthesis. In an *in vitro* experiment, lactic acid bacteria exerted effects on amino acid synthesis and metabolism [67]. However, the mechanisms involved in ZJ617 modulation of intestinal metabolism still need further investigation.

## Conclusion

The study revealed that ZJ617 could improve TJ protein expression related to the barrier function and suppress the activation of the MAPK and NF-κB inflammatory signaling pathways in the intestine of piglets challenged with LPS; interfer with intestinal and hepatic autophagy, apoptosis, and oxidative stress; and modulate the intestinal metabolism. These results suggest that ZJ617 pretreatment alleviates LPS-induced intestinal tight junction protein destruction, and intestinal and hepatic inflammatory and autophagy signal activation in piglets.

## Supplementary Information

**Additional file 1: **Supplementary Fig. S1. Ilea morphology, villus height and crypt depth of three groups. CON, control piglets orally inoculated with PBS; LPS, piglets treated by intraperitoneal injection (i.p.) of LPS (25 μg/kg body weight); ZJ617 + LPS, piglets orally inoculated with ZJ617 (1 × 10^10^ CFU/d) for 2 weeks before i.p. injection of LPS. The ileum tissue was stained with hematoxylin-eosin. Values are shown as mean ± SE, *n* = 6. Labeled means without a common letter differ, *P* < 0.05.

## Data Availability

The datasets generated for this study are available upon request to the corresponding author.

## Abbreviations

ALT: Alanine aminotransferase
AST: Aspartate aminotransferase
Atg5: Autophagy-related protein 5
Bax: B-cell lymphoma-2 associated X protein
DAO: Diamine oxidase
ERK: Extracellular signal-regulated kinase
FC: Fold change
GSH-Px: Glutathione peroxidase
I-κBα: Nuclear factor-kappa B inhibitor alpha
IL: Interleukin
JNK: c-Jun N-terminal kinase
KEGG: Kyoto Encyclopedia of Genes and Genomes
LC3: Microtubule-associated protein 1 light chain 3
LDH: Lactate dehydrogenase
LGG: *Lactobacillus rhamnosus* GG
LPS: Lipopolysaccharide
MAPK: Mitogen-activated protein kinase
MDA: Malondialdehyde
MPO: Myeloperoxidase
NF-кB: Nuclear factor-kappa B
OPLS-DA: Orthogonal projections to latent structures-discriminant analysis
PLS-DA: Partial least squares-discriminant analysis
RT: Retention time
SOD: Superoxide dismutase
TBA: Total bile acid
TNF-α: Tumor necrosis factor-α
VIP: Variable importance in projection
ZO: Zonula occludens
