# Supplementary material for: L. reuteri ZJ617 inhibits inflammatory and autophagy signaling pathways in gut-liver axis in piglet induced by lipopolysaccharide
Source: J Anim Sci Biotechnol. 2021 Oct 13;12:110. doi: 10.1186/s40104-021-00624-9 (PMC8513206; doi:10.1186/s40104-021-00624-9)
Supplement: Supplementary file 1 — Additional file 1: Supplementary Fig. S1. Ilea morphology, villus height and crypt depth of three groups. CON, control piglets orally inoculated with PBS; LPS, piglets treated by intraperitoneal injection (i.p.) of LPS (25 μg/kg body weight); ZJ617 + LPS, piglets orally inoculated with ZJ617 (1 × 10^10^ CFU/d) for 2 weeks before i.p. injection of LPS. The ileum tissue was stained with hematoxylin-eosin. Values are shown as mean ± SE, n = 6. Labeled means without a common letter differ, P < 0.05. [file 40104_2021_624_MOESM1_ESM.pdf]

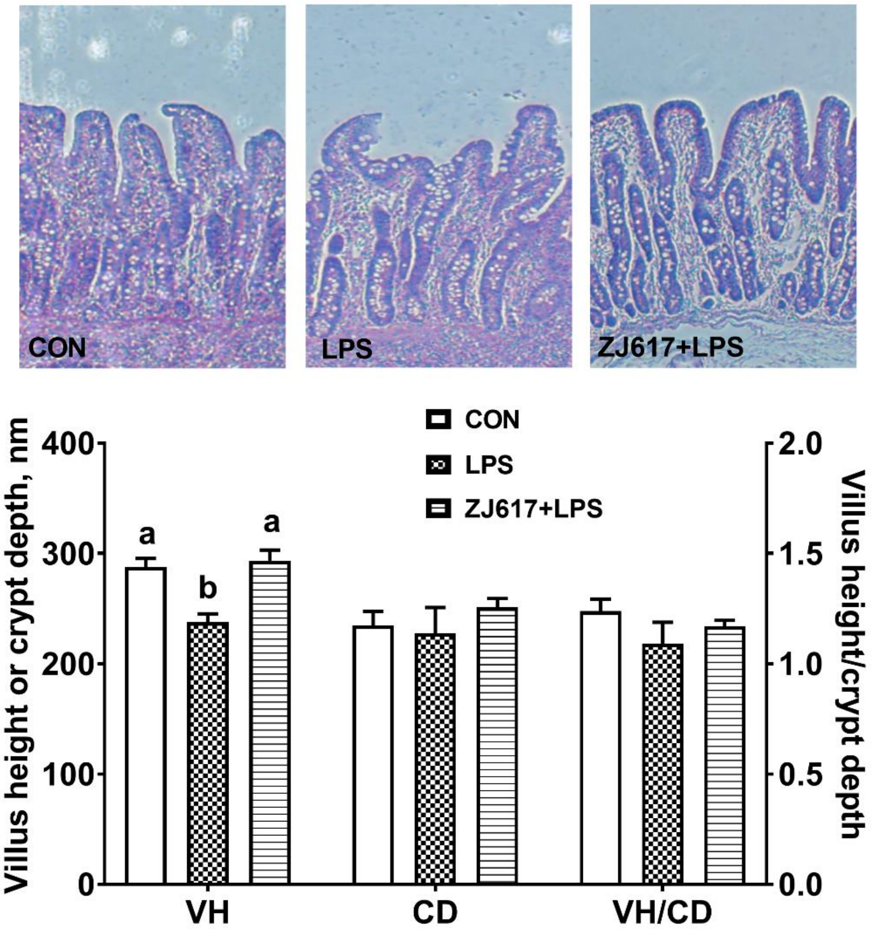

Supplementary Fig. S1. Ilea morphology, villus height and crypt depth of three groups. CON, control piglets orally inoculated with PBS; LPS, piglets treated by intraperitoneal injection (i.p.) of LPS (25 μg/kg body weight); ZJ617+LPS, piglets orally inoculated with ZJ617 (1×10^{10} CFU/d) for 2 weeks before i.p. injection of LPS. The ileum tissue was stained with hematoxylin-eosin. Values are shown as mean ± SE, n = 6. Labeled means without a common letter differ, $P < 0.05$.
